# Comparison of outcomes between novel oral anticoagulants and warfarin monotherapy in patients with left atrial appendage closure: A systematic review and meta-analysis

**DOI:** 10.3389/fcvm.2022.1023941

**Published:** 2022-10-24

**Authors:** Bing Sun, Rui Rui Chen, Chao Gao, Ling Tao

**Affiliations:** ^1^Department of Cardiology, Xijing Hospital, Air Force Medical University, Xi'an, China; ^2^Department of Cardiology, Tangdu Hospital, Air Force Medical University, Xi'an, China

**Keywords:** non-valvular atrial fibrillation, left atrial appendage closure, warfarin, novel oral anticoagulant, Device-related thrombus

## Abstract

**Background:**

Pivotal trials of percutaneous left atrial appendage closure (LAAC) used dedicated post-procedure antithrombotic protocols. However, there is no consensus on the selection of new oral anticoagulants (NOAC) and warfarin monotherapy after LAAC. This study aims to compare NOAC with warfarin monotherapy for efficacy and safety in patients undergoing LAAC.

**Methods:**

A database search was conducted using PubMed, EMBASE, Cochrane Library, and Clinicaltrials.gov for trials that compared NOAC with warfarin monotherapy after LAAC. The effective outcomes included any major adverse events (all-cause death, stroke, major bleeding) and their individual components. Safety outcomes included all-cause death, major bleeding, total bleeding, DRT, and PDL >5 mm.

**Results:**

We included 10 non-randomized clinical trials with 10,337 patients, of whom 4,960 patients received NOAC, while 5,377 patients received warfarin. There were no statistically significant differences in any major adverse events (LogOR: −0.11, 95% CI: −0.27, 0.04, *P* = 0.16), stroke (LogOR: 0.00, 95% CI: −0.42, 0.42, *P* = 1.00), all-cause death (LogOR: −0.23, 95% CI: −0.48, 0.02, *P* = 0.07), major bleeding (LogOR: −0.22, 95% CI: −0.45, 0.01, *P* = 0.06). NOAC was associated with a significant reduction in total bleeding (LogOR: −1.01, 95% CI: −1.47, −0.55, *P* < 0.0001) compared to warfarin. No statistically significant differences were found in DRT (LogOR: −0.19, 95% CI: −0.15, 0.52, *P* = 0.27) and PDL >5 mm (LogOR: 0.19, 95% CI: −0.33, 0.72, *P* = 0.47). Meta-regression and subgroup analysis showed that total bleeding (LogOR: −1.56, 95% CI: −2.15, −0.97, *P* < 0.001) was significantly lower in the NOAC group in the subgroup of <75 y.

**Conclusion:**

After LAAC, NOAC monotherapy was associated with a lower risk of bleeding compared to warfarin monotherapy for 45 days. There was no significant difference between NOAC and warfarin in terms of other results.

**Systematic review registration:**

www.york.ac.uk/inst/crd, identifier: CRD42022361244.

## Background

In patients with non-valvular atrial fibrillation, ischemic stroke and systemic embolism are believed to be associated with left atrial appendage (LAA) thrombi. Randomized controlled trials have shown that anticoagulation is effective in preventing stroke; however, many patients have contraindications to oral anticoagulation (1, 2). Several studies have reported that percutaneous closure of the left atrial appendix (LAAC) was not inferior to warfarin or NOAC for the prevention of thromboembolic events with additional reductions in major bleeding (3–6). In a larger-scale real-world study (7–9), the results have also confirmed the safety and efficacy of LAAC in stroke prevention.

As an implant, the healing process after implantation of a LAAC device is not fully understood. Limited animal studies in dogs showed that complete device endothelialization could last for 45 days (10). Therefore, in the crucial RCTs of LAAC, both the PROTECT AF and PREVAIL studies (3, 4) required patients to receive a combination of oral anticoagulation using warfarin plus aspirin for 45 days, followed by 6 months of dual platelet inhibition with clopidogrel and lifelong continuation of aspirin alone. However, both pre-LAAC planning and post-LAAC management have evolved over time, as there is a greater understanding of the peculiar and variable anatomy of the left atrial appendage (LAA) and how to balance the risk of bleeding and ischemic events. While warfarin has been widely used to prevent device-related complications (4–7), NOAC has been prescribed more frequently in contemporary studies (9, 11). Almost 66% of patients received new oral anticoagulants (NOAC) in the RECORD study (9), and all patients included in PINNACLE FLX were required to receive NOAC treatment at least 45 days of follow-up (11).

Previous meta-analysis have shown that NOAC was associated with significant reductions in stroke, intracranial hemorrhage, and mortality compared to warfarin in patients with atrial fibrillation (12). Theoretically, NOAC has fewer drug-drug interactions than warfarin and does not require a frequent blood draw to monitor the international normalized ratio (INR) (13). However, there is no consensus on the selection of novel oral anticoagulants (NOAC) and warfarin monotherapy after LAAC, and the results of clinical studies comparing the efficacy and safety of NOAC to warfarin monotherapy after LAAC were still inconclusive (14–23).

The current Expert Consensus Statement of the European Heart Rhythm Association/European Association for Cardio-Thoracic Surgery/European Society of Cardiology (EHRA/EAPCI/ESC) and the American College of Cardiology/American Heart Association (ACC/AHA) have recommended both medications (1, 2). The present study aims to systematically review and analyze the results of NOAC vs. warfarin monotherapy through meta-analysis after LAAC based on published research results to provide a basis for clinical medication guidance.

## Methods

We performed this systematic review and meta-analysis according to the PRISMA 2020 statement guideline (24). This study was registered at Prospero (CRD42022361244).

### Inclusion and exclusion criteria

The inclusion criteria are as follows: (1) Clinical trials that included patients who suffered from non-valvular atrial fibrillation (NVAF) with a high risk of stroke or bleeding and had successfully undergone LAAC treatment; (2) Studies reported any of efficacy or safety outcomes in patients who received NOAC vs. warfarin monotherapy for post-procedural anticoagulation after LAAC. The exclusion criteria are animal experiments, case reports, reviews, meta-analyses, conference proceedings without a full manuscript, and trials that did not directly compare NOAC vs. warfarin monotherapy were excluded.

### Intervention measures and results

After LAAC, NOAC or warfarin monotherapy was used for antithrombotic treatment. The efficacy outcomes included any major adverse events (all-cause death, stroke, major bleeding) and stroke. The safety outcomes were all-cause death, major bleeding, total bleeding, DRT, and PDL >5 mm. DRT and PDL >5 mm were documented by transesophageal echocardiography (TEE) evaluation at 45 days of follow-up.

### Search strategy

A database search was conducted using PubMed, EMBASE, Cochrane Library, and Clinicaltrials.gov using the keywords “atrial fibrillation, left atrial appendage closure; left atrial appendage occlusion; oral anticoagulant, novel oral anticoagulant, direct oral anticoagulant, NOAC, DOAC, non-Vitamin K antagonist anticoagulant, edoxaban, dabigatran, apixaban or rivaroxaban, Vitamin K antagonist, warfarin.” The duration of the retrieval was from the inception until July 1st, 2022.

### Selection process and data collection

We imported all references from the electronic search into Endnote 20 software and removed duplicates. The title and abstracts of the references were independently screened by two investigators to identify relevant studies. The same investigators then review the complete manuscripts of the relevant studies to determine eligibility for the study. In case of any disagreement, a third researcher would help to make the final decision. The extracted data contained: (1) name of the author and year of publication; (2) study design and sample size; (3) age, the score of CHA_2_DS_2_-VASC and HAS-BLED, type of NOAC, first TEE follow-up time, follow-up time. The Cochrane quality assessment tool was used in randomized controlled trials (RCT) (25), and the NOS scale was used in non-RCT studies (26). The high-quality literature was defined as 6–9 points as an overall score, the medium-quality literature was defined as 3–5 points, and the low-quality literature was defined as 1–2 points.

### Statistical methods

The odds ratio (OR) represented the effect index because the outcome index was a dichotomous variable, and point estimation of 95% CI was given for each index. Fixed continuity correction (addition of 0.5 to each cell) was used for trials with zero events. For every indicator, a point valuation or 95% CI was prescribed. We evaluated statistical heterogeneity using Cochran's *Q*-test (*P* < 0.05) and Higgins *I*^2^ statistics. The fixed effect model was used in the meta-analysis when *P* > 0.05 and *I*^2^ < 0.5. Publication bias was analyzed using the contour enhancement funnel plot and Egger's regression. Stata 17.0 (Stata Corp LLC) was used for statistical analyses. *P*-values < 0.05 were considered statistically significant.

## Results

### Screening of literature

**Figure 1:**
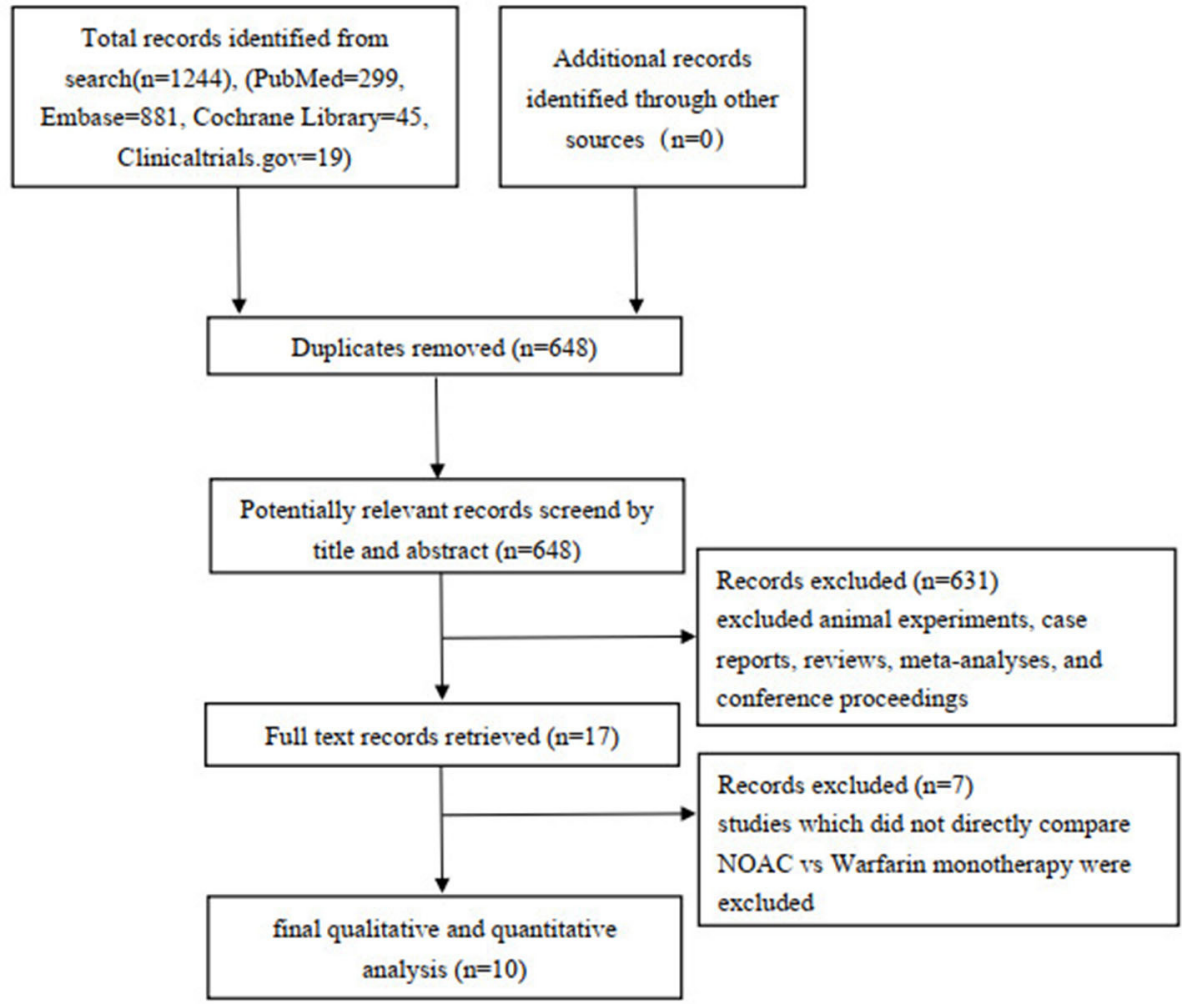
PRISMA flow diagram.

A total of 1,244 studies were retrieved from the PubMed, EMBASE, Cochrane Library, Clinicaltrials.gov databases, and 648 articles were selected after removing the duplicate. After evaluating the titles and abstracts, 631 articles were excluded (including reviews, animal experiments, meta-analyses, case reports, and meeting minutes), and seven articles were further excluded after reading the full text (7 articles did not directly compare NOAC vs. warfarin monotherapy regarding efficacy and safety outcomes). Finally, 10 articles were included in qualitative and quantitative studies (Figure 1).

### Study characteristics

The 10 included studies were all non-RCTs published between 2016 and 2022. Four trials had a follow-up time of 45 days, and the others had a follow-up duration for 3 months to 12 months. The first transesophageal echocardiogram (TEE) follow-up was performed at 45 days. In these trials, different types of NOAC were used, including dabigatran, rivaroxaban, apixaban, and edoxaban. The mean age <75 years was in six trials, the mean CHA2DS2-VASC ≥4 was exhibited in six trials, and HAS-BLED was found to be ≥3 in five trials. The post-implant antithrombotic strategy was to give NOAC or warfarin monotherapy for 45 days and dual antiplatelet therapy (DAPT) with aspirin and clopidogrel for 3–6 months. Amplatzer Cardiac Plug 13.8% (47/340) was used only in the study of Chen et al. (18), and others applied the WATCHMAN device. Some other factors, such as a history of stroke and previous bleeding, types of atrial fibrillation (AF), and left ventricular ejection fraction (LVEF), were not provided in nearly half of all included studies. Therefore, these data were not recorded. The study characteristics are presented in Table 1. The quality assessment of the included literature studies was assessed using the NOS scales (Supplementary Table 1).

**Table 1:** Baseline data and procedural characteristics.

**References**	**Design**	**Age**		**Sample size**		**CHA**_**2**_ **DS**_**2**_ **-VAS**_**C**_		**HAS-BLED**		**Types of NOAC**	**The first TEE performs**	**Follow-up time**
		**NOAC**	**Warfarin**	**NOAC**	**Warfarin**	**NOAC**	**Warfarin**	**NOAC**	**Warfarin**			
Enomoto (14)	Non-RCT	76 ± 8	75 ± 8	214	212	3.8 ± 1.4	4.1 ± 1.4	2.4 ± 1.0	2.7 ± 0.9	Dabigatran (7%), Rivaroxaban (46.1%), Apixaban (45.8%), Edoxaban (1%)	6 weeks	4 months
Bergmann et al. (15)	Non-RCT	73.4		109	155	4.1	4	1.9	2.1	Dabigatran (43.1%), Rivaroxaban (35.8%), Apixaban (21.1%)	<3 months	3 months
Cohen et al. (16)	Non-RCT	76 ± 7.5	77 ± 8	47	43	4.7 ± 1.5	4.7 ± 1.5	3.5 ± 0.8	3.5 ± 1	Dabigatran (NR), Rivaroxaban (NR), Apixaban (87.2%)	6 weeks	8 months
Adedinseow (17)	Non-RCT	77		52	162	5.0 (2.0–8.0)		4.0 (1–7)		NR	6 weeks	6 weeks
Fu et al. (19)	Non-RCT	70	70	291	77	4.6 ± 1.5 4.5 ± 1.6	4.5 ± 1.4	3.0 ± 1.0 2.9 ± 1.0	3.1 ± 1.0	Dabigatran (56.7%), Rivaroxaban (43.3%)	45 days	45 days
Zhu et al. (20)	Non-RCT	67	65	40	30	4.05 ± 1.09	3.0 ± 1.37	3.18 ± 0.59	3.17 ± 0.82	Dabigatran (40%), Rivaroxaban (60%)	45 days	45 days
Chen et al. (18)	Non-RCT	65 ± 7.7	64 ± 8.2	164	170	3.3 ± 1.6	2.9 ± 1.5	1.9 ± 1.1	1.7 ± 1.2	NR	45 days	45 days
Freeman et al. (21)	Non-RCT	76 ± 8	76 ± 8	3948	4330	4.50 ± 1.47	4.45 ± 1.45	2.85 ± 1.13	2.75 ± 1.14	NR	45 days	6 months
Ajmal et al. (23)	Non-RCT	75.3 ± 7.0	74.3 ± 8.0	57	152	4 (3–4)	4 (3–5)	4 (3–4)	4 (3–4)	Dabigatran (7%), Rivaroxaban (40.4%), Apixaban (49.1%), Edoxaban (1%)	45 days	1 year
Ge et al. (22)	Non-RCT	66.1 ±	10.9 69.8 ± 8.5	38	46	3.8 ± 1.4	4.1 ± 1.6	2.7 ± 0.8	2.7 ± 1.1	Dabigatran (100%)	6 weeks	12 months

### Outcomes

#### Effective outcomes

**Figure 2:**
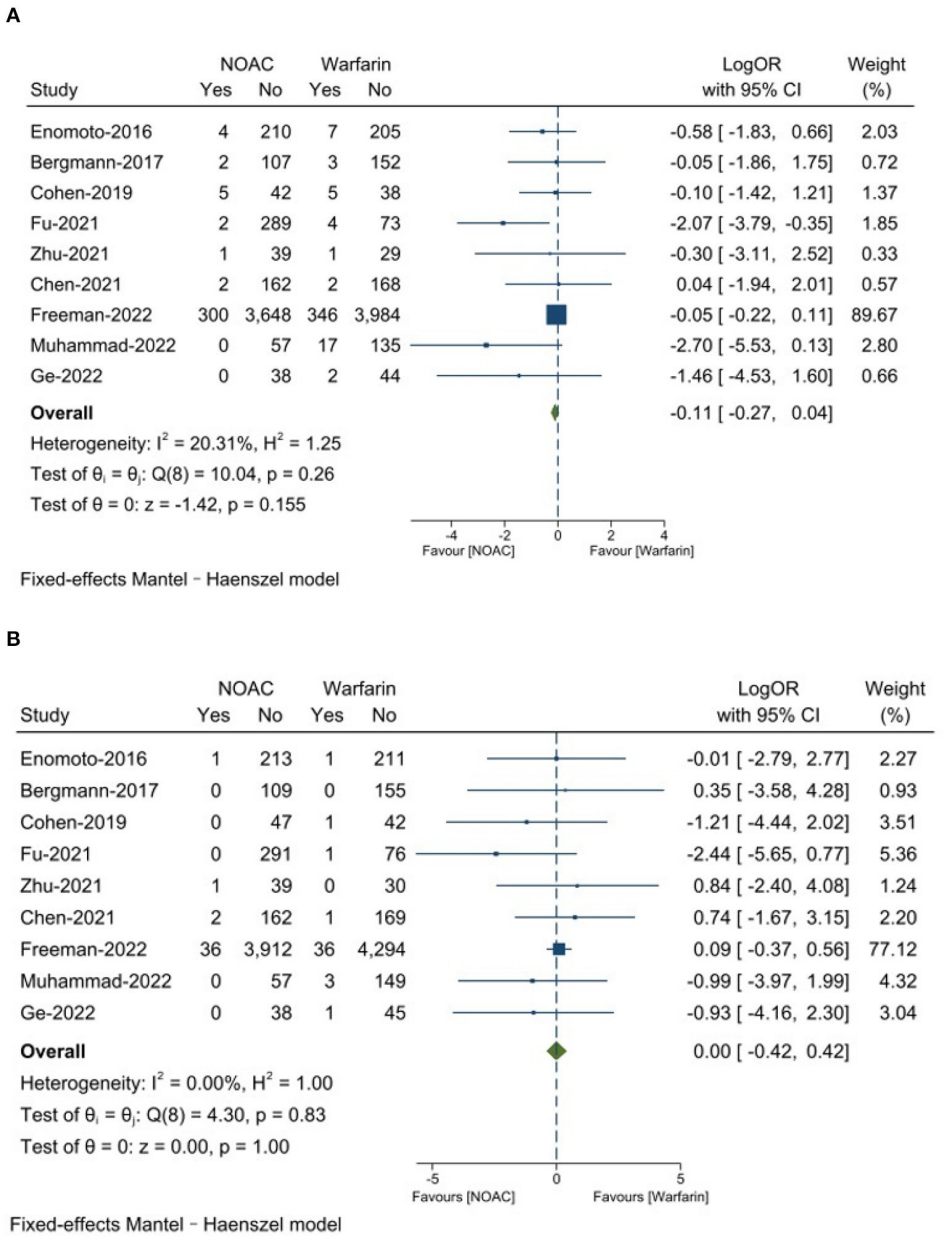
**(A)** Any major adverse events; **(B)** Stroke.

There was no significantly significant differences on any major adverse event between NOAC group 6.4% and warfarin group 7.4% (LogOR: −0.11, 95% CI: −0.27, 0.04, *P* = 0.16). The NOAC group had 0.8% strokes and the warfarin group had 0.8% (LogOR: 0.00, 95% CI: −0.42, 0.42, *P* = 1.00). The results are presented in Figures 2A,B.

#### Safety outcomes

**Figure 3:**
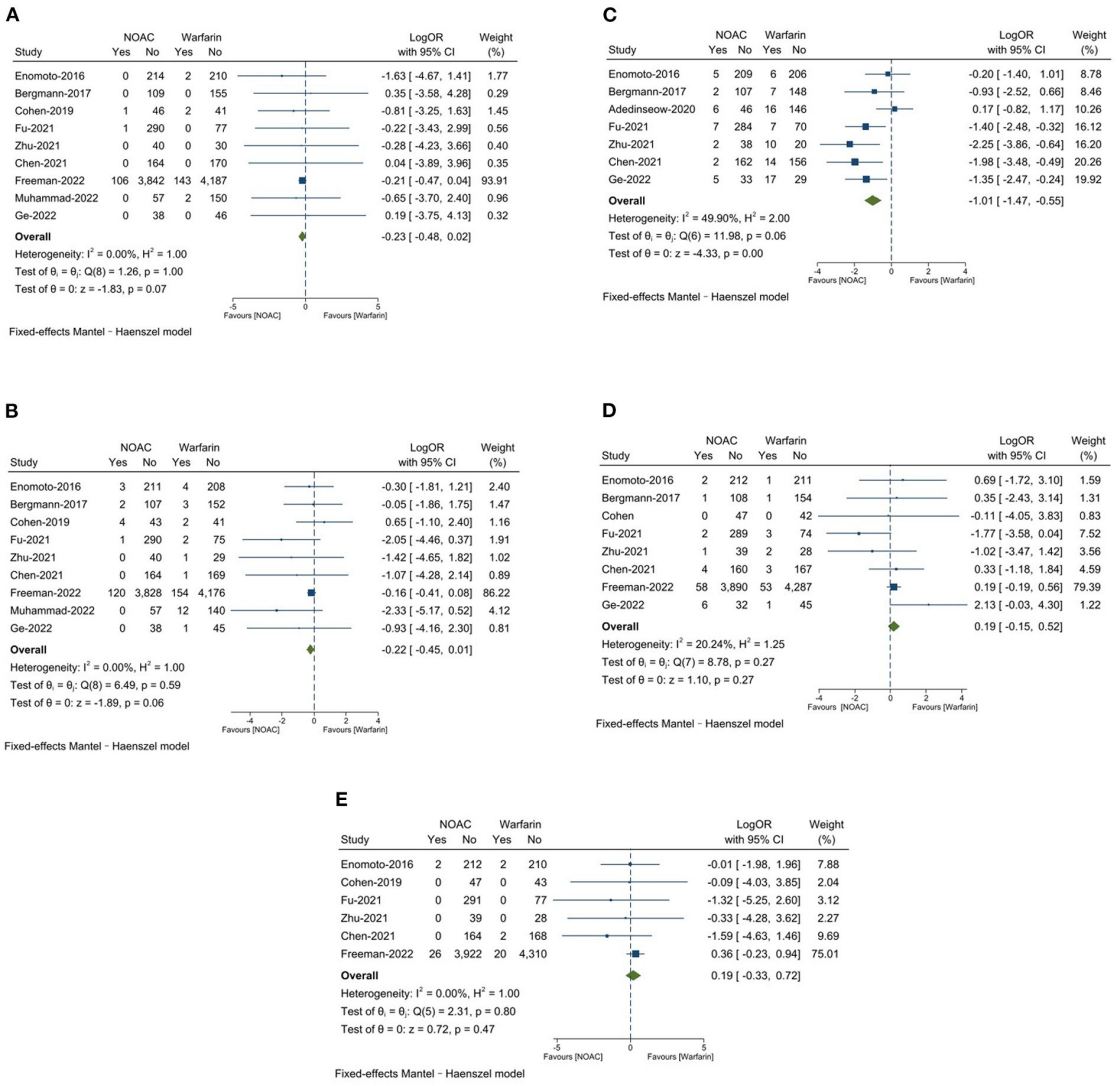
**(A)** All-cause death; **(B)** Major bleeding; **(C)** Total bleeding; **(D)** Device-related thrombus (DRT); **(E)** Peri-device leaks (PDL >5 mm).

There was no significantly significant difference on all-cause death between NOAC group 2.2% and warfarin group 2.9% (LogOR: −0.23, 95% CI: −0.48, 0.02, *P* = 0.07). The major bleeding was found to be 2.6% in the NOAC group and 3.5% in the warfarin group (LogOR: −0.22, 95% CI: −0.45, 0.01, *P* = 0.06), the differences was not significantly significant. The NOAC group had 3.2% of total bleeding, which was significantly lower than the warfarin group with 9.0% (LogOR: −1.01, 95% CI: −1.47, −0.55, *P* < 0.0001). DRT was 1.5% in the NOAC group and 1.2% in the warfarin group 1.2% (OR: −0.19, 95% CI: −0.15, 0.52, *P* = 0.27), the differences was not significantly significant. The NOAC group had 0.6% of PDL >5 mm, which was numerically similar to the warfarin group 0.5% (OR: 0.19, 95% CI: −0.33, 0.72, *P* = 0.47). Results are presented in Figures 3A–E.

#### Subgroup analysis and meta-regression

The NOAC group was significantly lower any major adverse events than the warfarin group in the subgroup of <75 years (LogOR: −1.20, 95% CI: −2.04, −0.36, *P* = 0.005), HAS-BLED ≥3 (LogOR: −1.20, 95% CI: −2.06, −0.34, *P* = 0.006), and the test of the group difference was statistically significant (*P* = 0.01, *P* = 0.01). No significant differences were found about stroke between groups in the subgroup of follow-up time, age, CHA2DS2-VASC, and HAS-BLED. No significant group differences had been found in all-cause death by follow-up time, age, CHA2DS2-VASC, and HAS-BLED. The NOAC group had significantly lower bleeding than the warfarin group in the subgroup <75 years (LogOR: −1.25, 95% CI: −2.28, −0.23, *P* = 0.017), and the group difference test was statistically significant (*P* = 0.04). The NOAC group had significantly lower total bleeding than the warfarin group in the subgroup <75 years (LogOR: −1.56, 95% CI: −2.15, −0.97, *P* < 0.001), and the group difference test was statistically significant (*P* < 0.001). The mean HAS-BLED was associated with DRT, and the group difference test was statistically significant (*P* = 0.03), but no statistically significant differences were found between the two groups in the HAS-BLED subgroup. No significant group differences were found on PDL >5 mm of follow-up time, age, CHA2DS2-VASC, and HAS-BLED. Based on the results of the subgroup analysis, we made a meta-regression and found that mean age was a predictor of total bleeding. The results are presented in Supplementary Figures 1a–g.

#### Sensitivity analysis

The leave-one-out sensitivity analyses were used by Stata 17.0. Freeman-2022 represented 76% of the sample size of our study. When Freeman et al. was excluded (21), there was a statistically significant difference in the outcome of any major adverse event (LogOR: −0.84, 95% CI: −1.44, −0.24, *P* = 0.006). In the sensitivity analysis of major bleeding, excluding Cohen et al. (16), the result was a statistically significant difference between the two groups (LogOR: −0.24, 95% CI: −0.47, −0.01, *P* = 0.045). The results of the sensitivity analysis of stroke, DRT, total bleeding, and PDL >5 mm remained stable compared to the main analysis. The results are presented in Supplementary Figures 2a–g.

#### Publication bias

**Figure 4:**
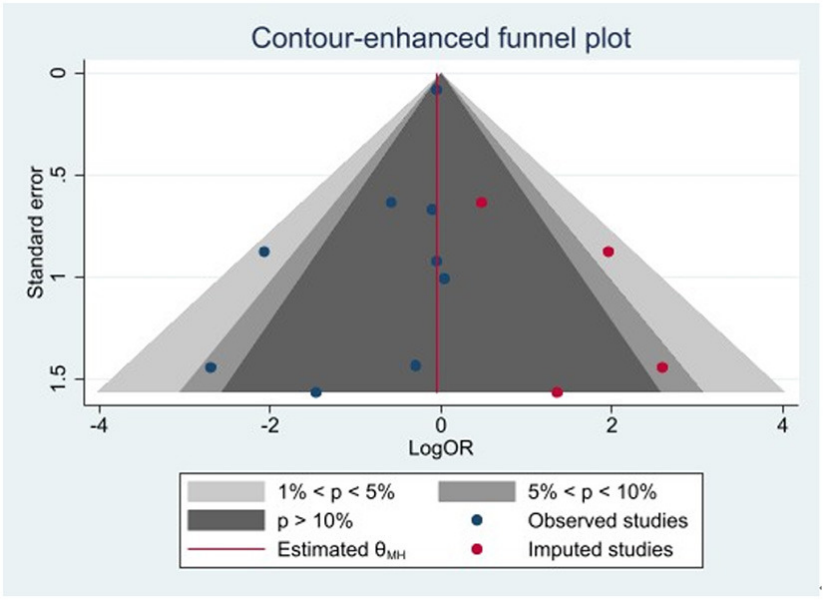
Publication bias.

Publication bias was analyzed for any major adverse events using the contour enhancement funnel plot and shear complement method. Imputed studies obtained = 4, and the result of Egger's regression was *p* = 0.037. Our study had a major publication bias, and there may be studies with unpublished negative results. The results are presented in Figure 4.

## Discussion

The main findings of our study can be summarized as follows.

1. There were no statistical differences between NOAC and warfarin in terms of the risk of major adverse events or stroke.
2. NOAC was not associated with a lower risk of all-cause death and major bleeding compared to warfarin, while total bleeding was significantly lower in the NOAC group than in the warfarin group.
3. There were no statistical differences between NOAC and warfarin with respect to the risk of DRT and PDL >5 mm at the first TEE follow-up.
4. Patients with age <75 years were associated with a lower risk of total bleeding while using NOAC than warfarin after the LAAC procedure.

We noted that patients receiving NOAC had a numerically lower rate of all-cause death (2.2 vs. 2.9%) compared to warfarin. Previous meta-analysis has shown that NOAC was associated with a lower risk of intracranial bleeding, hemorrhagic stroke, and fatal bleeding than warfarin (12). In our study, the lower trend of all-cause death in the NOAC group should be associated with lower major bleeding (2.8 vs. 3.5%) and total bleeding (4.3 vs. 9.1%) compared to the warfarin group. The low-dose of NOAC could be a reason for the decrease in the risk of bleeding compared to warfarin (27). In Ge-2022, low-dose dabigatran (110 mg twice daily) was associated with significantly lower rates of bleeding compared to warfarin (13.2 vs. 34.8%, *P* = 0.02) (22). Warfarin is more likely to cause recurrent bleeding than NOAC in patients with a history of gastrointestinal bleeding (28). In our study, nearly 45% of the patients had a history of gastrointestinal bleeding (21), which could contribute to an elevated level of bleeding in the warfarin group compared to the NOAC group. However, no study is designed to explore the relationship between mortality and bleeding in patients post-LAAC. More real-world data are needed to assess the causality between death and bleeding. Furthermore, younger patients (<75 y) derived a lower risk of bleeding from NOAC than warfarin, which was consistent with the existing study (29). In particular, our results showed that NOAC still significantly reduced the risk of bleeding with short-term anticoagulation of 45 days compared to warfarin after LAAC, which was different from long-term anticoagulation in previous studies.

Although DRT is rare, it was associated with a 3-fold higher risk of stroke and a systemic embolism (30). The incidence of DRT in this study was 1.5% in the NOAC group and 1.2% in the warfarin group, which was 3–5% less than in the mentioned studies. The occurrence of DRT from 90 days to 1 year was considered to be about 58% of the total number of DRT events (31). Therefore, the short-term follow-up time in our studies resulted in a lower incidence of DRT. The risk of DRT tended to be higher in the NOAC group than in the warfarin group (1.5 vs. 1.2%), which may be due to the different types and low doses of NOAC. It seems to be superior to dabigatran to prevent DRT after LAAC (32). Rivaroxaban effectively reduced the occurrence of DRT after the LAAC procedure better than dabigatran, possibly because dabigatran increased platelet aggregation, thus increasing the risk of DRT (33). Low-dose dabigatran (110 mg twice a day) was associated with a higher risk of DRT at 45 days compared to warfarin (22). However, these studies had small sample sizes, and more data is needed to verify these conclusions. PDL is also identified as a potential risk factor for major adverse events (34, 35); however, it is not associated with the anticoagulation strategy, according to our results. The current study reported PDL >5 mm was similar between the two groups (0.6 vs. 0.5%). This discrepancy in the geometry of the LAA and the device may lead to incomplete LAA occlusion and residual leaks, and the device compression rate of <10% may be the predictor of PDL (36).

The new generation Watchman FLX device is a new option for further applications. Watchman FLX achieved near 100% implantation success and a substantial decrease in the occurrence of periprocedural complications compared to Amplatzer Amulet occlusion (11, 37). Watchman FLX also had a lower DRT at 45 days (38) and a higher sealing rate at 3 months compared to the Watchman device in a small sample size of clinical trials (39). Larger RCTs are needed to evaluate the superiority of Watchman FLX.

Compared with previous meta-analysis comparing NOAC vs. warfarin after LAAC (40), the current report has included several updates. First, we included three additional studies meeting our criteria (17, 21, 22) (Freeman-2022, Ge-2022, and Adedinsewo-2020) and excluded (41–43), which compared NOAC+ASA *vs*. warfarin +ASA (41–43) instead of NOAC *vs*. warfarin monotherapy. Second, our study had a larger sample size of 10,796 patients compared to 2,440 patients in the previous study. Third, we performed a meta-analysis comprised of any major adverse events and stroke, as compared to previous study that investigated separately. In addition, meta-regressions and subgroup analyses of follow-up time, age, CHA_2_DS_2_-VASc, and HAS-BLED scores were also performed to identify the subgroup of patients who were prone to favor NOAC or warfarin. We found that in patients who had an age <75 y, NOAC might be associated with better clinical outcomes compared to warfarin.

### Limitations

This study also had the following limitations: (1) there was a major publication bias in our study, which could have influenced the credibility of the results; (2) the antithrombotic regimen and duration of administration after LAAC and follow-up time were changed, and the definitions of major bleeding were also different in the literature. These factors provide underlying sources of clinical heterogeneity in the meta-analysis. The Higgins *I*^2^ statistics in the pooled analysis outcomes were <50%; (3) We were unable to obtain all individual data for a subgroup analysis, such as a history of stroke and prior bleeding, types of AF and left ventricular ejection fraction (LVEF). These risk factors, which could have affected our results, were not reported in some of the included studies; (4) All included studies are non-RCTs, with intrinsic limitations, including the risk of selection bias, confounding bias, and inability to attribute causality. We need a larger RCT to verify these results; (5) The results of the subgroup analysis can only be a potential trend rather than definite conclusions based on the average value of factors.

## Conclusions

According to the results of the present study, new oral anticoagulants (NOAC) are similar to warfarin in preventing major adverse events and stroke in the follow-up period of 45 days-12 months after LAAC. NOAC reduced all-cause death more than warfarin, which may be associated with a lower risk of major and total bleeding. Due to the observational nature of included studies, the results in the study are regarded as generated hypotheses. The results should be verified through randomized trials. Many patients after LAAC tend to suffer bleeding, but optimal timing and antithrombotic therapy strategies remain unknown (7). Thus, more RCTs should be conducted to test the current results.

## Data availability statement

The original contributions presented in the study are included in the article/Supplementary material, further inquiries can be directed to the corresponding author/s.

## Author contributions

BS: implementation research, analyze and interpret data, and article writing. RC: statistical analysis. CG: incubation, design of experiments, and make a critical review of the intellectual content of the article. LT: design of experiments and obtaining research funding. All authors contributed to the article and approved the submitted version.

## Funding

This study was supported by the Program for National Science Funds of China (Grant No. 82170358).

## Conflict of interest

The authors declare that the research was conducted in the absence of any commercial or financial relationships that could be construed as a potential conflict of interest.

## Publisher's note

All claims expressed in this article are solely those of the authors and do not necessarily represent those of their affiliated organizations, or those of the publisher, the editors and the reviewers. Any product that may be evaluated in this article, or claim that may be made by its manufacturer, is not guaranteed or endorsed by the publisher.
