# Supplementary material for: Comparison of outcomes between novel oral anticoagulants and warfarin monotherapy in patients with left atrial appendage closure: A systematic review and meta-analysis
Source: Front Cardiovasc Med. 2022 Oct 24;9:1023941. doi: 10.3389/fcvm.2022.1023941 (PMC9639703; doi:10.3389/fcvm.2022.1023941)
Supplement: Supplementary file 1 [file Data_Sheet_1.pdf]

Supplementary Material

Supplementary Figure 1a. Subgroup of any major adverse event

Supplementary Figure 1b. Subgroup of stroke

Supplementary Figure 1c. Subgroup of all-cause death

Supplementary Figure 1d. Subgroup of major bleeding

Supplementary Figure 1e. Subgroup of total bleeding

Supplementary Figure 1f. Subgroup of device-related thrombus (DRT)

Supplementary Figure 1g. Subgroup of peri-device leaks (PDL>5mm)

Supplementary Figure 2a. Sensitivity analysis of any major adverse event

Supplementary Figure 2b. Sensitivity analysis of stroke

Supplementary Figure 2c. Sensitivity analysis of all-cause death

Supplementary Figure 2d. Sensitivity analysis of major bleeding

Supplementary Figure 2e. Sensitivity analysis of total bleeding

Supplementary Figure 2f. Sensitivity analysis of device-related thrombus (DRT)

Supplementary Figure 2g. Sensitivity analysis of peri-device leaks (PDL>5mm)

Supplementary Table 1. Quality assessment

Supplementary Figure 1a. Subgroup of any major adverse events

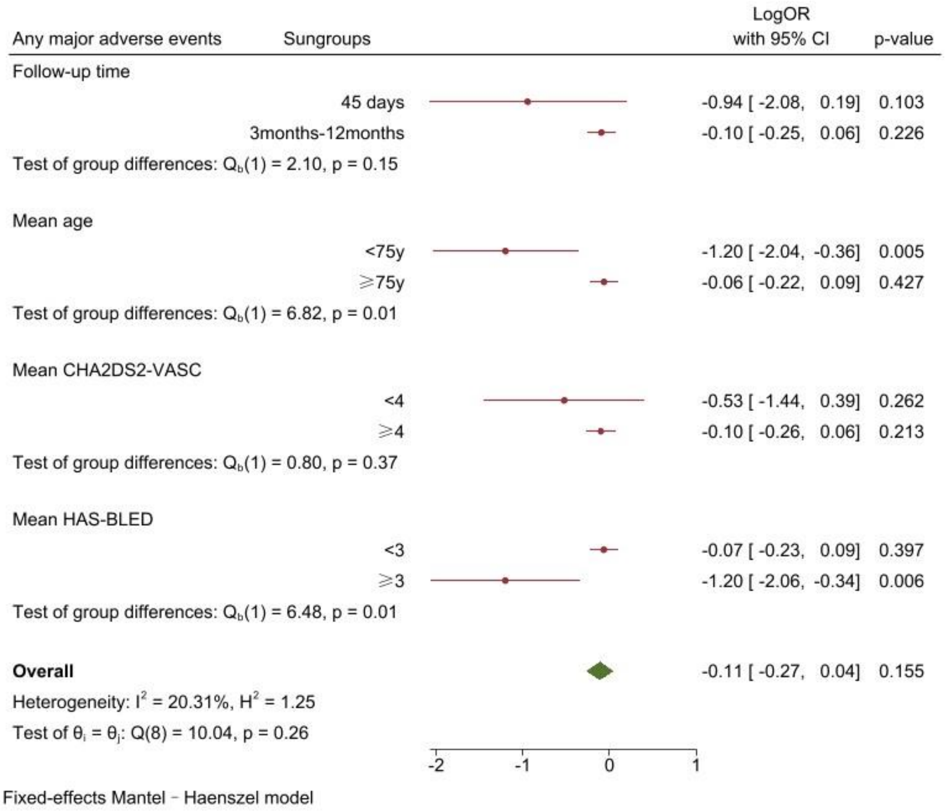

Supplementary Figure 1b. Subgroup of stroke

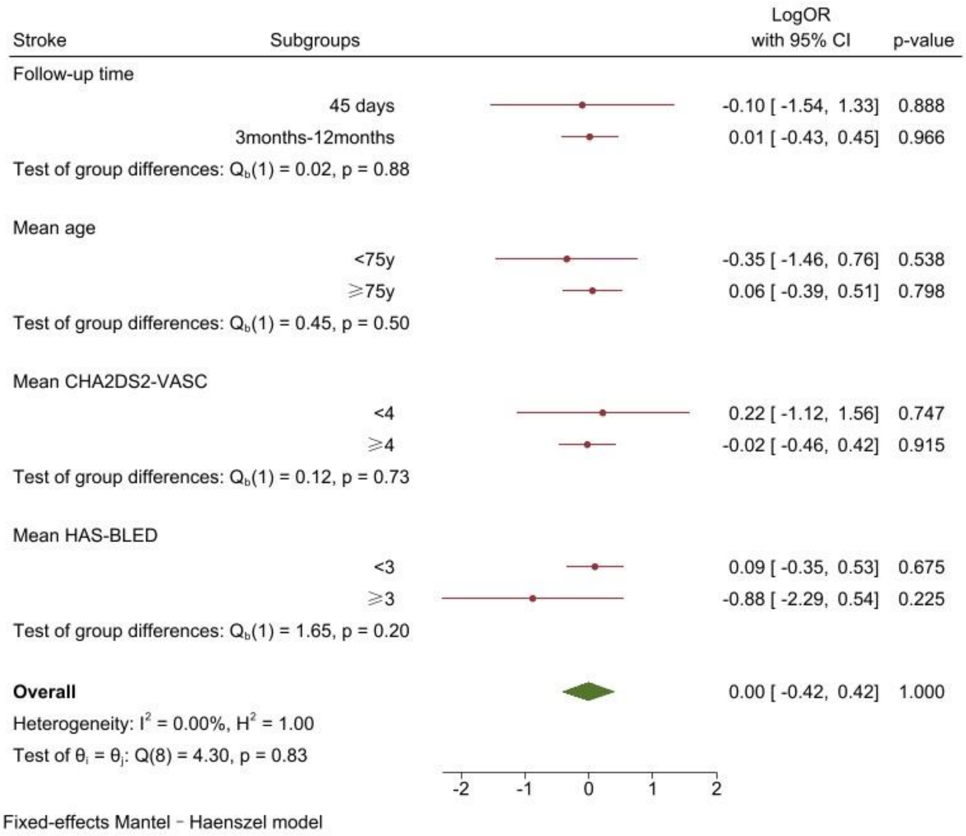

Supplementary Figure 1c. Subgroup of all-cause death

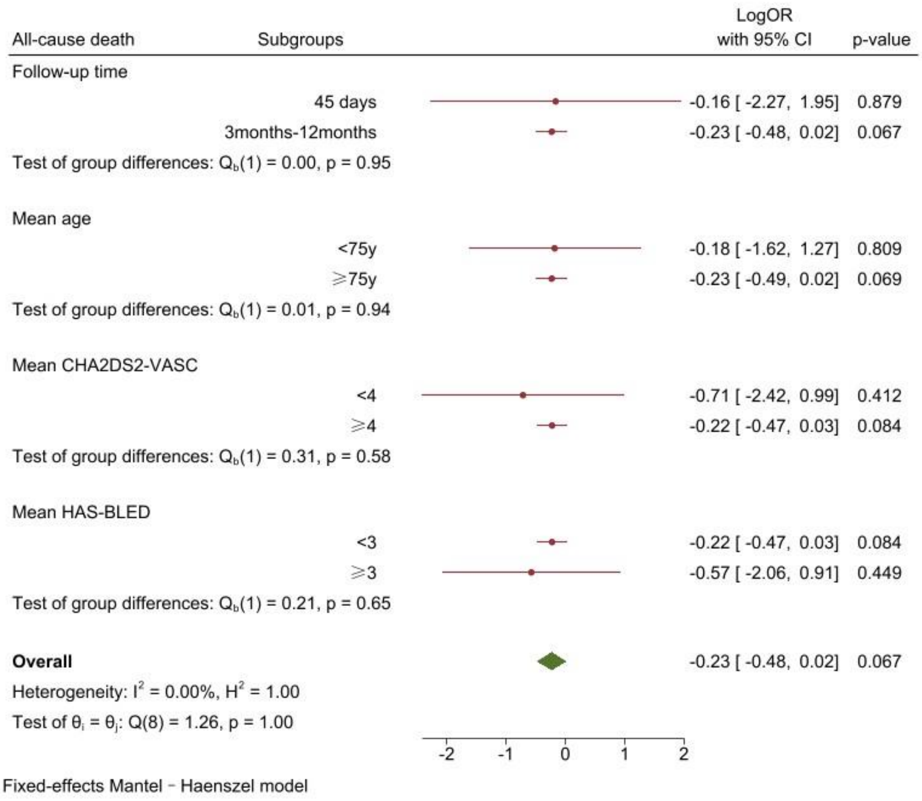

Supplementary Figure 1d. Subgroup of major bleeding

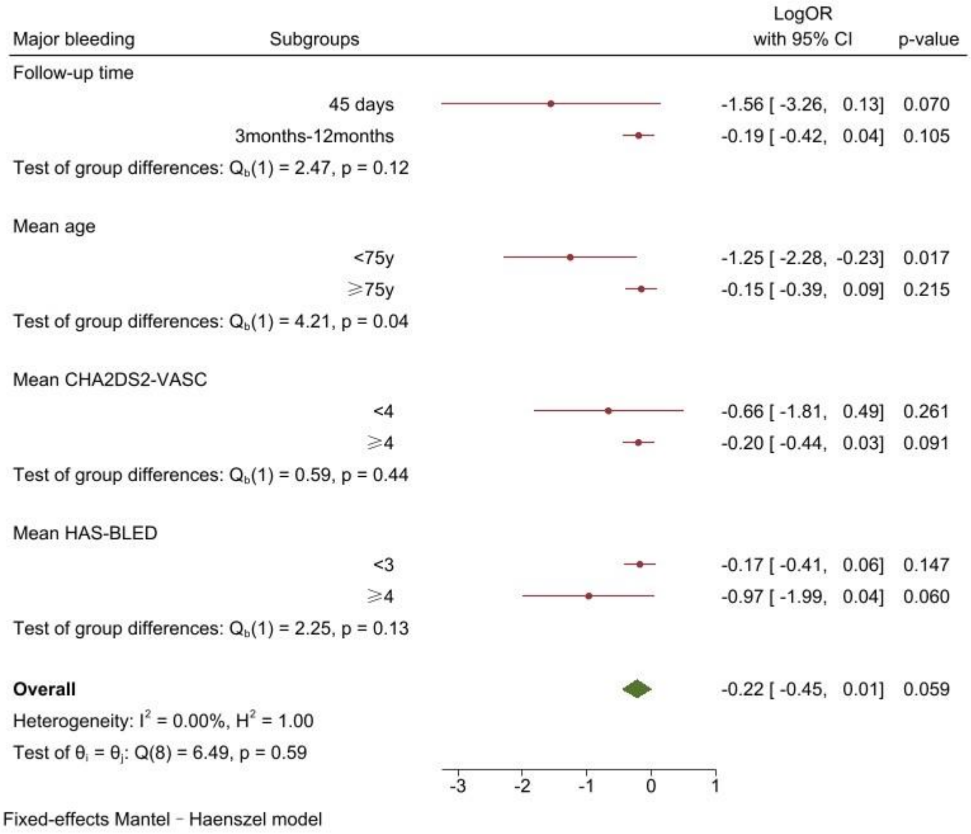

Supplementary Figure 1e. Subgroup of total bleeding

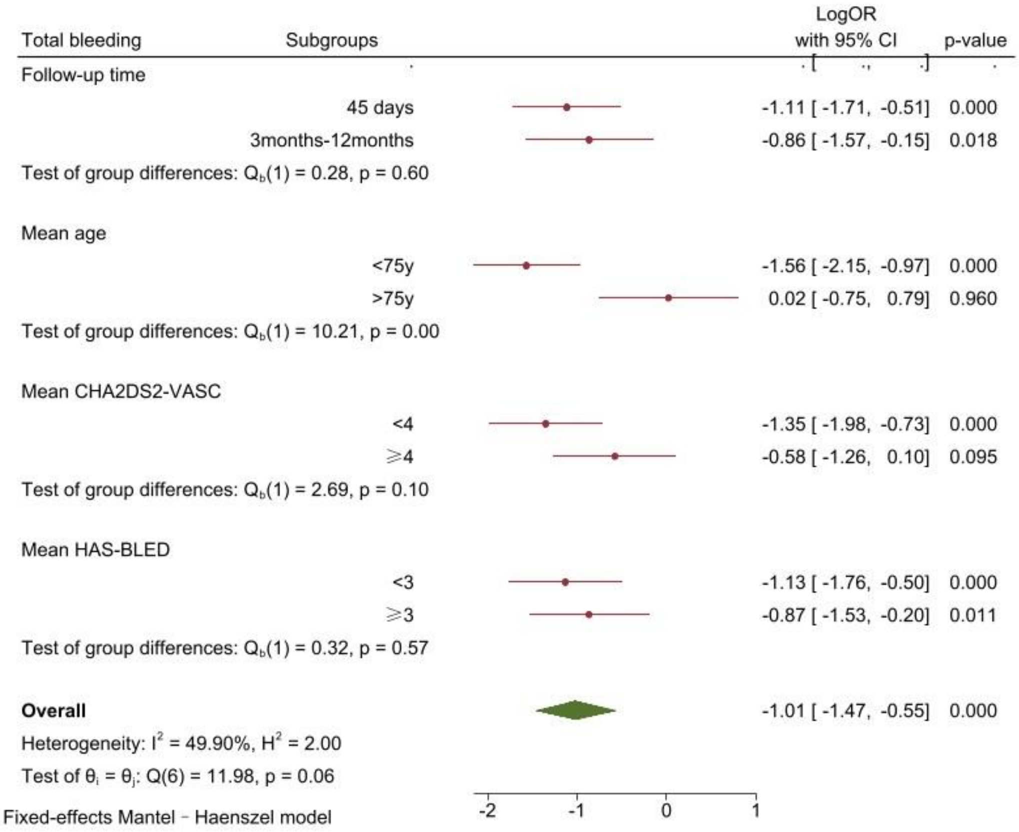

Supplementary Figure 1f. Subgroup of device-related thrombus (DRT)

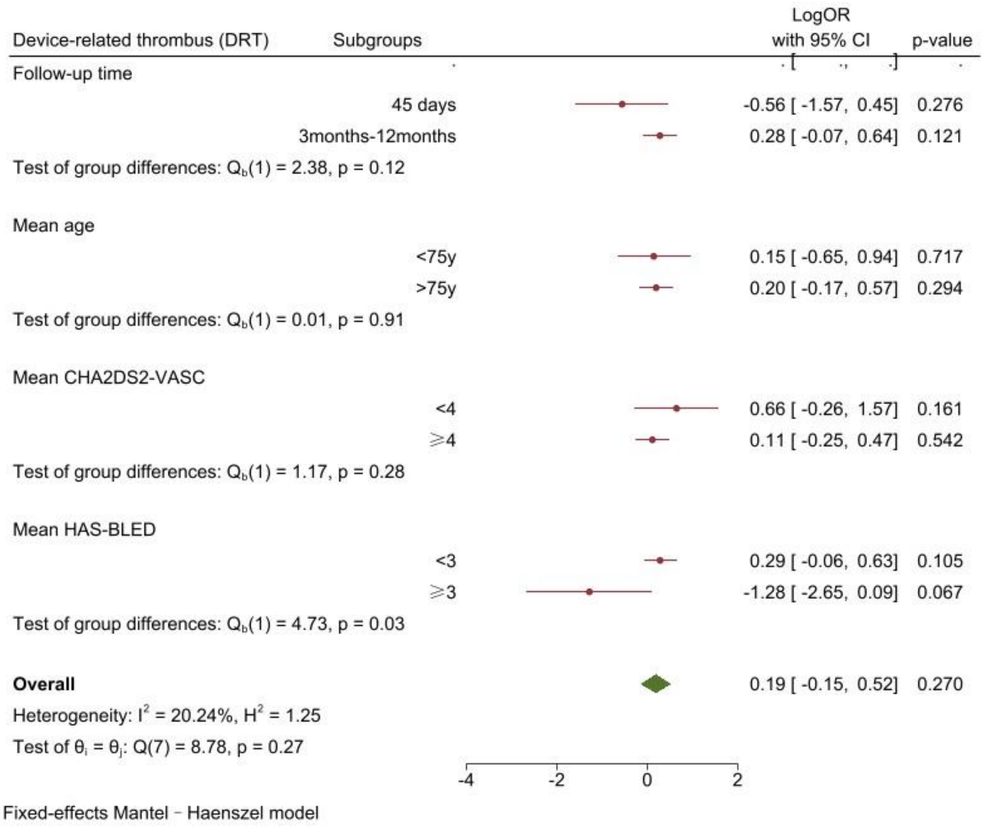

Supplementary Figure 1g. Subgroup of peri-device leaks (PDL>5mm)

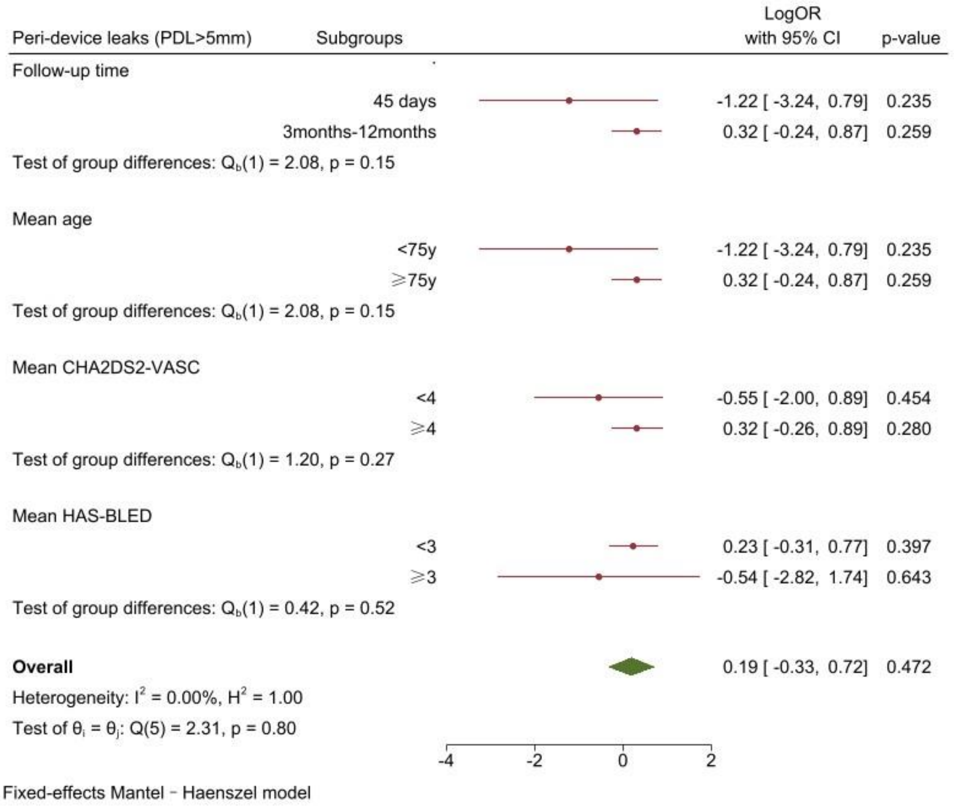

Supplementary Figure 2a. Sensitivity analysis of any major adverse events

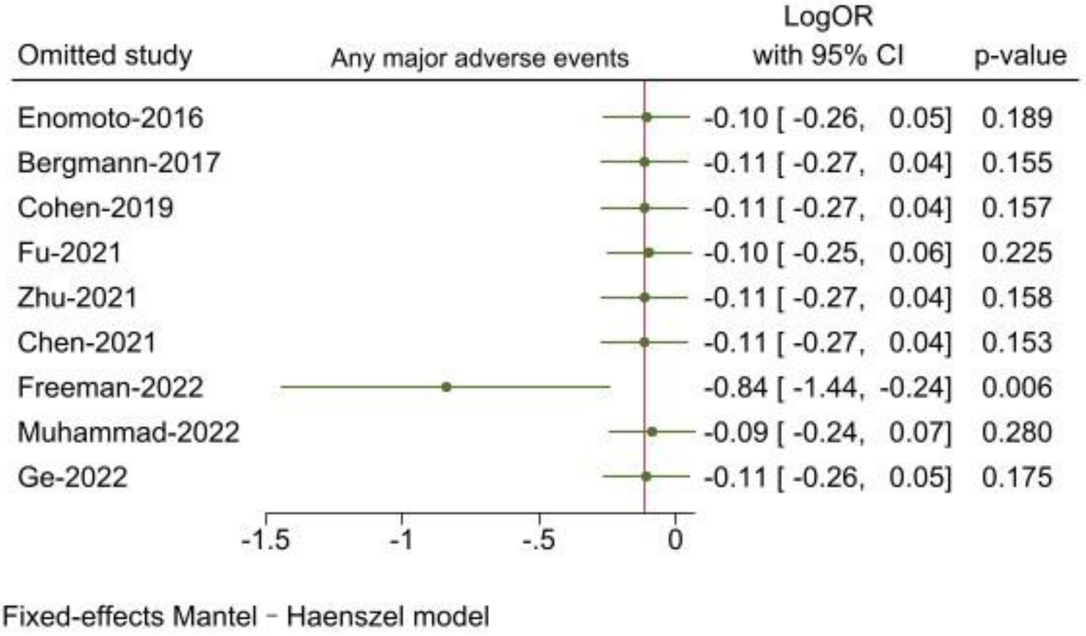

Supplementary Figure 2b. Sensitivity analysis of stroke

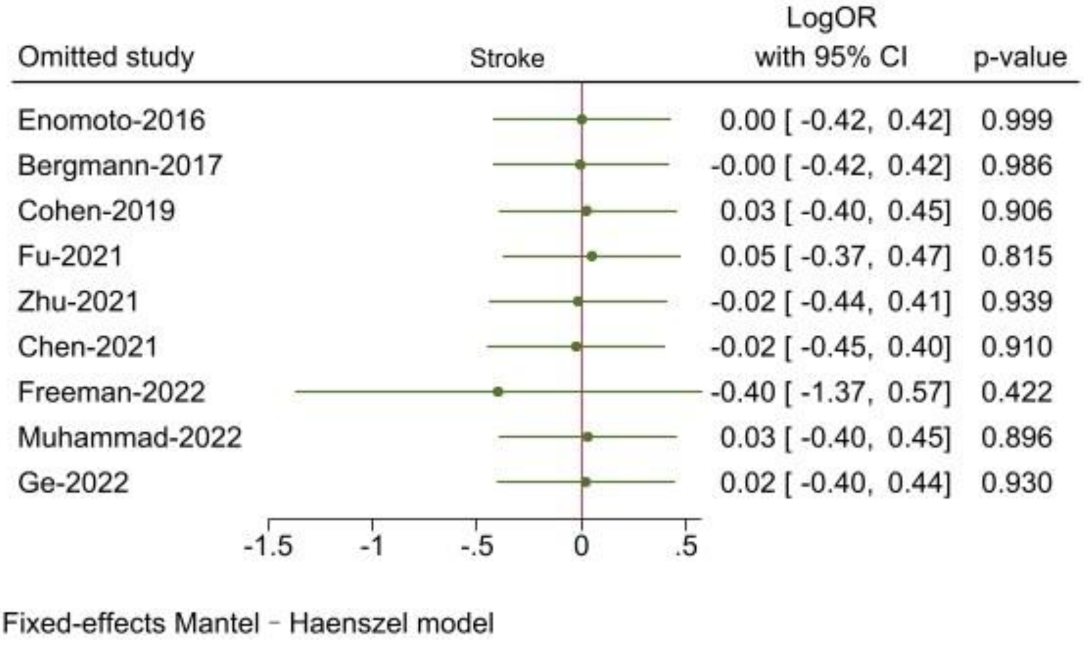

Supplementary Figure 2c. Sensitivity analysis of all-cause death

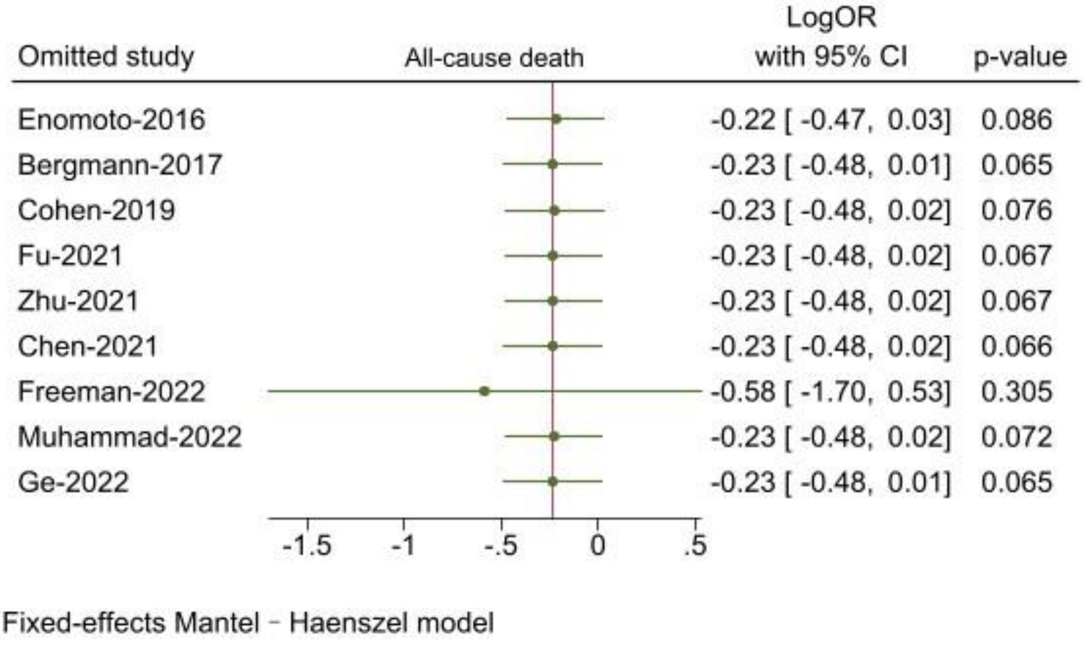

Supplementary Figure 2d. Sensitivity analysis of major bleeding

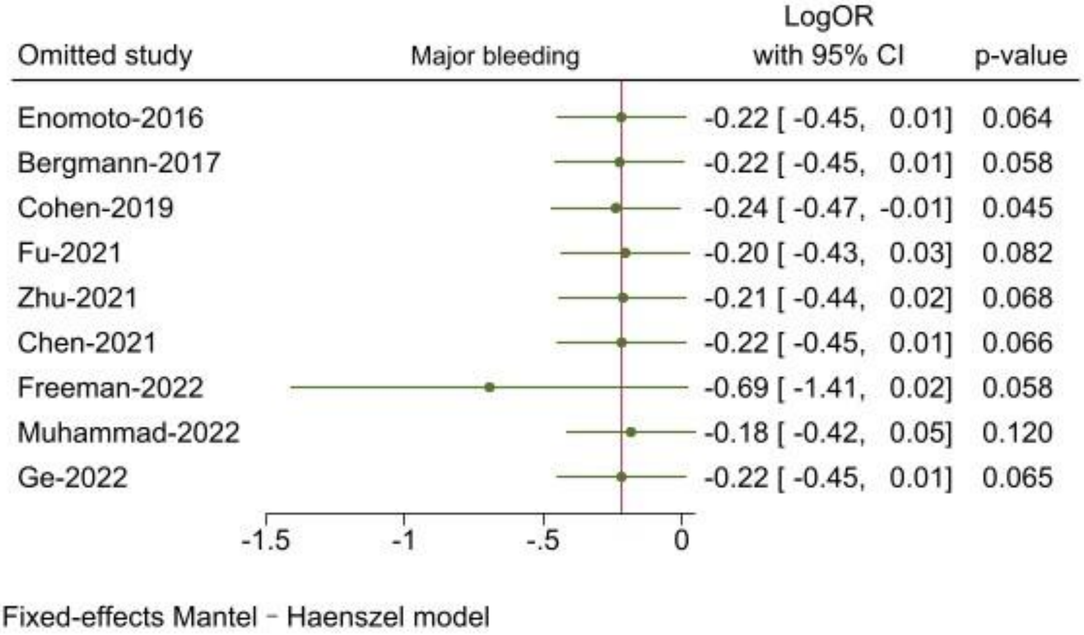

Supplementary Figure 2e. Sensitivity analysis of total bleeding

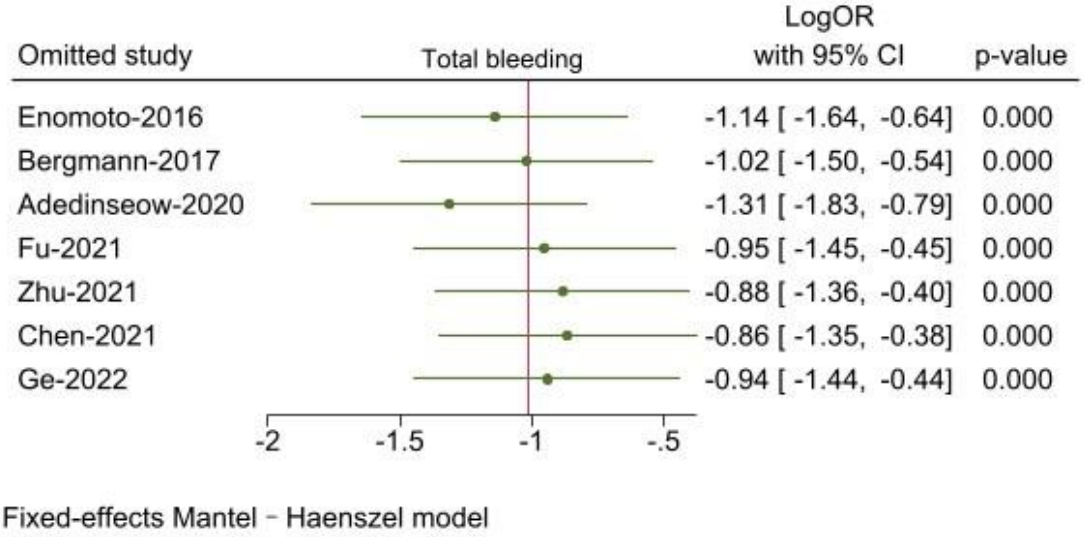

Supplementary Figure 2f. Sensitivity analysis of device-related thrombus (DRT)

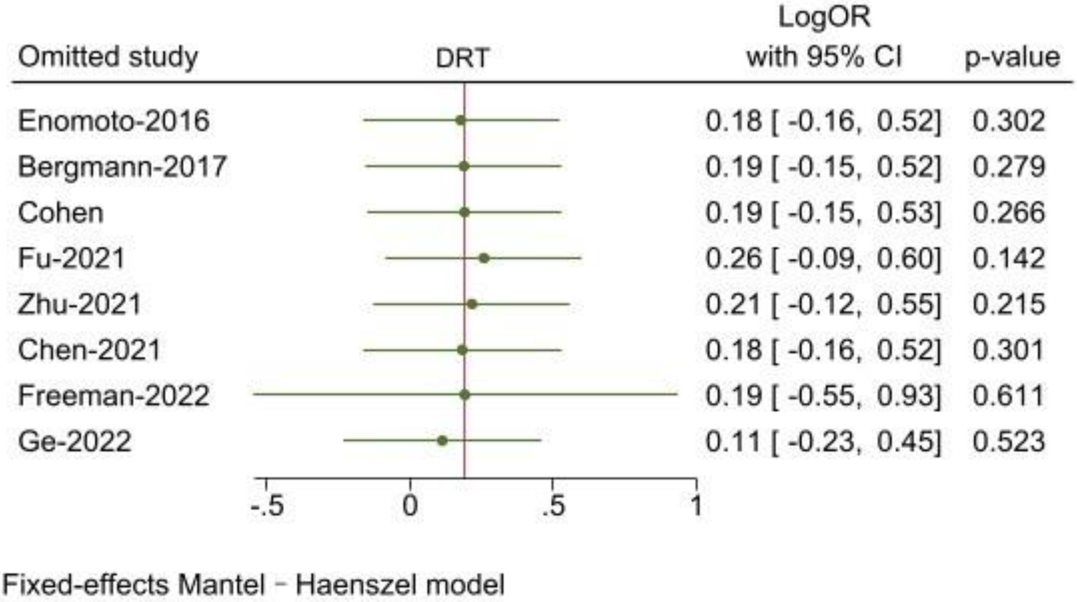

Supplementary Figure 2g. Sensitivity analysis of peri-device leaks (PDL>5mm)

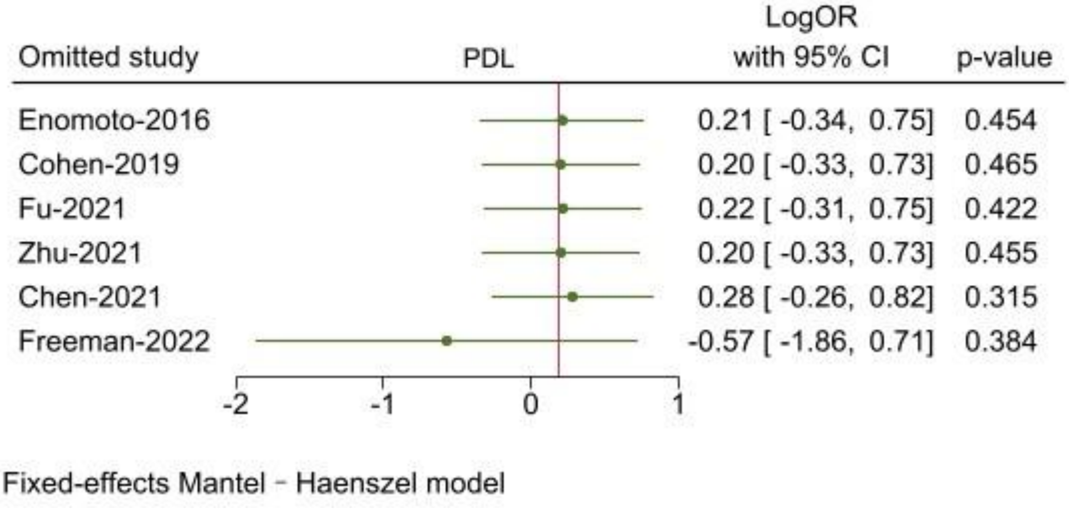

Supplementary Table 1. Quality assessment

Author and year	Selection of case (4 scores)				Comparison between Definition and diagnosis of cases (2 scores)	methods of exposure assessment (4 scores)		
	Definition and diagnosis of cases	Representativeness of cases	selection of controls	Definition of controls		Representativeness of cases (2 scores)	Same method of ascertainment for cases and controls	Investigation No response rates
Enomoto-2016	√	√	√	√	√√	√	√	√
Bergmann-2017	√	√	√	√	√√	√	√	√
Cohen-2019	√	√	√	√	√√	√	√	√
Adedinseow-2020	√			√	√			√
Fu-2021	√	√	√	√	√√	√	√	√
Zhu-2021	√	√	√	√	√	√	√	√
Chen-2021	√	√	√	√	√√	√	√	√
Freeman-2022	√	√		√	√		√	√
Ge-2022	√	√	√	√	√	√		√
Muhammad-2022	√	√	√	√	√	√		√
